# Integrating Spatiotemporal Epidemiology, Eco-Phylogenetics, and Distributional Ecology to Assess West Nile Disease Risk in Horses

**DOI:** 10.3390/v13091811

**Published:** 2021-09-12

**Authors:** John M. Humphreys, Angela M. Pelzel-McCluskey, Lee W. Cohnstaedt, Bethany L. McGregor, Kathryn A. Hanley, Amy R. Hudson, Katherine I. Young, Dannele Peck, Luis L. Rodriguez, Debra P. C. Peters

**Affiliations:** 1 Pest Management Research Unit, Agricultural Research Service, US Department of Agriculture, Sidney, MT 59270, USA; 2 Veterinary Services, Animal and Plant Health Inspection Service (APHIS), US Department of Agriculture, Fort Collins, CO 80526, USA; Angela.M.Pelzel-McCluskey@usda.gov; 3 Arthropod-Borne Animal Disease Research Unit, Agricultural Research Service, US Department of Agriculture, Manhattan, KS 66502, USA; Lee.Cohnstaedt@usda.gov (L.W.C.); Bethany.McGregor@usda.gov (B.L.M.); 4 Department of Biology, New Mexico State University, Las Cruces, NM 88003, USA; khanley@nmsu.edu (K.A.H.); Kiy761@nmsu.edu (K.I.Y.); 5 Big Data Initiative and SCINet Program for Scientific Computing, Agricultural Research Service, US Department of Agriculture, Beltsville, MD 20704, USA; amy.hudson@usda.gov (A.R.H.); Deb.Peters@usda.gov (D.P.C.P.); 6 Northern Plains Climate Hub, US Department of Agriculture, Fort Collins, CO 80526, USA; Dannele.Peck@usda.gov; 7 Plum Island Animal Disease Center, US Department of Agriculture, Orient Point, NY 11957, USA; Luis.Rodriguez@usda.gov

**Keywords:** West Nile virus, horses, equine, mosquito, eco-phylogenetics, avian reservoir, spatial non-stationarity, disease biogeography, Bayesian

## Abstract

Mosquito-borne West Nile virus (WNV) is the causative agent of West Nile disease in humans, horses, and some bird species. Since the initial introduction of WNV to the United States (US), approximately 30,000 horses have been impacted by West Nile neurologic disease and hundreds of additional horses are infected each year. Research describing the drivers of West Nile disease in horses is greatly needed to better anticipate the spatial and temporal extent of disease risk, improve disease surveillance, and alleviate future economic impacts to the equine industry and private horse owners. To help meet this need, we integrated techniques from spatiotemporal epidemiology, eco-phylogenetics, and distributional ecology to assess West Nile disease risk in horses throughout the contiguous US. Our integrated approach considered horse abundance and virus exposure, vector and host distributions, and a variety of extrinsic climatic, socio-economic, and environmental risk factors. Birds are WNV reservoir hosts, and therefore we quantified avian host community dynamics across the continental US to show intra-annual variability in host phylogenetic structure and demonstrate host phylodiversity as a mechanism for virus amplification in time and virus dilution in space. We identified drought as a potential amplifier of virus transmission and demonstrated the importance of accounting for spatial non-stationarity when quantifying interaction between disease risk and meteorological influences such as temperature and precipitation. Our results delineated the timing and location of several areas at high risk of West Nile disease and can be used to prioritize vaccination programs and optimize virus surveillance and monitoring.

## 1. Introduction

Mosquito-borne West Nile virus (WNV) is the causative agent of West Nile disease in humans, horses, and some bird species [1,2,3]. The virus is a member of the Flaviviridae family and belongs to the same sero-group as the arthropod-borne viruses (arboviruses) Japanese encephalitis virus, Usutu virus, Murray Valley encephalitis virus, and St. Louis encephalitis virus [4,5,6,7]. WNV is the most common cause of neuroinvasive arboviral disease in the contiguous US: the average human incidence was recently estimated by Curren et al. [8] to be 0.44 cases/100,000 persons and we [9] calculated estimates approximately 10% higher at 0.48 cases/100,000 after accounting for uneven reporting and environmental risk factors. Less is known about risk factors contributing to WNV infection of horses. In the years immediately following the 1999 US introduction of WNV, equine WNV vaccines were rapidly developed and licensed [10,11]: however, despite the initial post-invasion push to reduce what can be substantial economic impacts to horse owners, there has yet to be a large-scale, comprehensive analysis of equine West Nile disease (WND) in the US [12,13,14]. Research describing the abiotic and biotic drivers of equine WND is needed to anticipate the spatial and temporal distribution of disease risk, improve disease surveillance, and avoid economic impacts to the agricultural industry and private horse owners.

Since introduction in 1999, WNV has infected more than 27,000 horses in the US, with mortality rates estimated between 30 and 50% and neurologic symptoms that include stumbling, aimless wandering, convulsions, inability to swallow, impaired vision, teeth grinding, hind limb weakness, paralysis, and coma [15,16,17,18,19]. Due to research funding reductions over the past decade, there have been calls to designate WND as a neglected disease, but given its recent US introduction, WND is generally considered an emerging disease in the US and a re-emerging disease globally [14,20,21,22].

WNV was originally described from Uganda in 1937 [23] and then subsequently identified at locations throughout Africa, Asia, and Europe over the next half-century, though the virus was not considered a serious equine health or economic issue until the mid-1990s [14,24]. Prior to the mid-1990s, equine seropositivity rates as high as 54% were reported from Northern Africa (1950s) and isolated horse epizootics with mortality were documented in Europe (1962–1963). Because such epizootics were infrequent, WNV was not perceived as major threat to agriculture [24,25]. Perceptions changed in the 1990s when virus reemergence resulted in a marked increase in the number and severity of equine WNV infections worldwide and coincided with virus introduction and spread in the Western Hemisphere [26,27,28,29]. A 1996 outbreak in Morocco affected 94 horses, of which 45% died; 58% equine seroprevalence was estimated in herds near Tuscany (Italy) in 1998 after 14 horses from the region displayed neurologic symptoms; 75 horses were infected in Israel between 1998 and 2000, of which 20% died; and France confirmed 76 infected horses in 2000 after more than 130 displayed signs of disease [24,30,31]. In the Western Hemisphere, 20 horses were infected in New York during the year of initial virus introduction (1999), with 63 more confirmed equine cases across the Northeastern US in 2000 [32,33]. Between 2000 and 2005, WNV rapidly spread across the Western Hemisphere with serologic evidence or confirmed neurologic disease reported in horses from all states in the contiguous US as well as Canada, the Caribbean, Mexico, Central America, and South America [28,34,35]. Given the extraordinary rapidity with which WNV can move across large distances and impact agriculture, the equine industry, and private horse owners, studies to elucidate the drivers of enzootic transmission are urgently needed to assess prevailing risk and to forecast future disease outbreaks.

Although WNV readily infects horses, they are “dead-end” hosts [36] that do not contribute to forward transmission. The virus is maintained in cycles involving ornithophilic mosquito vectors (primarily species within the genus *Culex*) and avian reservoir host species [2]. Thus, we applied a disease biogeography approach to analyze spatiotemporal relationships among four major WNV system components: (1) horse hosts, (2) mosquito vectors, (3) avian reservoir hosts, and (4) extrinsic climatic, socio-economic, and environmental factors. Disease biogeography leverages quantitative methods common to distributional ecology to investigate infectious disease from an integrated ecological and epidemiological perspective [37,38]. Our aim was to assess equine WND risk from the ecological-epidemiology perspective that risk is dependent on prevailing rates of equine WNV infection and the location-specific exposure of horses to WNV, as well as the complex network of biotic and abiotic environmental factors that mediate reservoir and vector spatiotemporal distributions. To this end, we estimated the “absolute” and “relative” risk of equine WND, where absolute risk was defined as the total number of cases predicted for a given time, location, and horse population (abundance) and relative risk was the ratio of absolute risk to the expected case number based on disease rates for the larger US over the period of record.

We place our analysis within the disease biogeography paradigm because our framework was derived from human epidemiology methods with risk estimates contingent on (horse) population incidence and disease exposure rather than environmental suitability, occurrence probability, or abundance as is standard for niche models [38,39,40,41]. This distinction is important to understand technical aspects of model statistical implementation and is central to interpreting WNV ecology. In contrast to pathogens such as avian influenza virus that may be transmitted by both environmental and avian reservoirs [42,43,44], free WNV (outside of host or vector bodies) is not known to significantly contribute to disease propagation, and therefore the geographic distribution of WNV is likely little restricted by abiotic environmental conditions beyond those that shape host and vector species occurrence. Stated differently, the WNV “niche” is better described by host and vector availability, competency, and community assembly than it is by the climate or edaphic conditions external to these organisms. Because WNV nidality was assumed to be a reflection of highly-mobile avian host availability [45], we adopted a modern niche concept [46] that expanded on inclusion of the abiotic environment (*Grinnellian niche*), pathogen interactions with hosts and vectors (*Eltonian niche*), and the pathogen’s fundamental niche (*Hutchinsonian niche*) to quantify shifts in virus distributions due to access and transport by competent avian hosts [41,47].

Avian species in the Order Passeriformes serve as the principal WNV reservoirs [48,49,50,51,52]. Passeriformes (perching birds) are the largest and most diverse clade of birds in the world, show variable WNV competence, and exhibit a wide range of long-distance and local dispersal behaviors making selection of any one species as a representative or archetypal virus reservoir problematic [53,54]. Further complicating analysis, pathogen hosts do not function in isolation and are instead embedded in a local community with interacting organisms (hosts and non-hosts) that is, in turn, nested within a larger host metacommunity at the landscape scale. The nested, hierarchical structure of pathogen–host interactions gives rise to cross-scale dynamics that influence prevalence at the local scale (county-scale) and transmission processes at the landscape scale (continental-scale) as communities are bridged by reservoir migration and dispersal [55,56]. Techniques from phylogenetic community ecology (eco-phylogenetics) have potential to untangle cross-scale transmission dynamics in the WNV system. Eco-phylogenetics represent the merger of community ecology with phylogenetics and are increasingly being used to investigate host–parasite and disease systems [57,58,59].

Although predicting risk of disease transmission in multi-host systems is an outstanding challenge in infectious disease ecology, an improved understanding of the evolutionary and phylogenetic aspects of host community assembly and composition may help garner insight into virus dilution and amplification effects at the community and landscape scales [56,59]. Virus dilution and amplification effects in relation to host diversity have been proposed to shape WNV transmission [60,61,62,63,64]. Our objective was to explain WND biogeography and to assess horse WND risk across the US by integrating spatiotemporal epidemiology, eco-phylogenetics, and distributional ecology.

## 2. Materials and Methods

### 2.1. Study Area and Disease Data

Our study area encompassed the contiguous US which includes a geographic extent greater than 9.8 million km${}^{2}$. Equine WND incidence data were acquired from the Centers for Disease Control and Prevention (CDC) [65] as a text file. Tabulated data provided the number of confirmed horse WND cases reported within each US county between 2009 and 2018. We used the reported case onset date to aggregate case counts to occurrence month. Cases documented between 2009 and 2017 were used for model training and those reported in 2018 were partitioned for out-of-sample model validation.

### 2.2. Virus Surveillance

The number of sentinel animal, avian, and mosquito WNV detections reported to the CDC were also obtained for the study period (CDC, 2019). ArboNET is a passive surveillance system. It is dependent on clinicians considering the diagnosis of an arboviral disease and obtaining the appropriate diagnostic test, and reporting of laboratory-confirmed cases to public health authorities. Diagnosis and reporting are incomplete, and the incidence of arboviral diseases is underestimated. We combined these virus detection reports and then aggregated to the county and month of detection to create a WNV surveillance covariate.

To help account for variation in county surveillance and reporting, we generalized (spatial and temporally smoothed) the WNV surveillance covariate by estimating virus detection probability for each county and month using the following formula,

(1) $$\begin{array}{c} p_{st}^{v}∼\mathrm{binomial}\left(\pi_{st}^{v}\right)\\ \mathrm{logit}\left(\pi_{st}^{v}\right)=\xi_{s}^{v}+\gamma_{t}^{v} \end{array}$$

where the probability of virus detection ($p_{st}^{v}$) in county *s* and time *t* (month of year) followed a binomial likelihood with a mean $\pi_{st}^{v}$ and a response variable coded as,

$$p_{st}^{v}=\left\{\begin{array}{cc} 1, & \mathrm{if}\;\mathrm{virus}\;\mathrm{detected}\;\mathrm{in}\;\mathrm{county},\;\mathrm{and}\\ 0, & \mathrm{otherwise}. \end{array}\right.$$

The $\xi_{s}^{v}$ term shown in Equation (1) symbolizes a spatial covariate constructed using a Besag–York–Mollié (BYM) configuration (Besag et al., 1991) that included scaling between structured and unstructured BYM components to improve estimates [66,67,68,69]. The $\xi_{s}^{v}$ covariate quantified spatial dependencies between counties based on a neighborhood graph (adjacency matrix), which we constructed using the spdep package [70], with contiguity based on a “queen” configuration (only one adjoining point needed to define a neighbor). We found that the 3109 counties in the US had between 1 and 14 neighbors, with an average of 5.78 adjoining neighbors per county. This same neighborhood graph was also used for disease modeling decribed in Section 2.6. A monthly time trend ($\gamma_{t}^{v}$) was added using a first-order (one time-step) random walk (curvilinear response) defined as $\gamma_{t}=\gamma_{t-1}+\Delta \gamma_{t}$, with the current time step based on the prior step plus an incremental $\Delta \gamma_{t}$, where $\Delta \gamma_{t}=N\left(0,\sigma^{2}\right)$ and included an enforced sum to zero constraint (centered on zero).

### 2.3. Climate Data

Mean monthly maximum temperature and total precipitation data for the US (2009–2018) were acquired from the PRISM Climate Group at a 4 km grid resolution [71] using the prism package [72]. Monthly mean temperature and precipitation estimates were then averaged by county.

Weekly drought indices reporting the areal proportion of each county subject to six different drought stages were obtained from the US DroughtMonitor (https://droughtmonitor.unl.edu/) (accessed on 2 July 2021) and averaged to monthly values. The US Drought Monitor produces categorical drought stages based on a combination of metrics: the Palmer Drought Severity Index, NOAA’s Climate Prediction Center Soil Moisture Model percentiles, USGS Weekly Streamflow percentiles, Standardized Precipitation Index, and expert opinion. Arranged from the least to most dry stage, the reported drought levels included No Drought, Abnormally Dry, Moderate Drought, Severe Drought, Extreme Drought, and Exceptional Drought.

### 2.4. Avian Host Occurrence, Prevalence, and Phylogenetic Data

Several model covariates were developed to evaluate relationships between avian WNV host species and equine WND occurrence. To develop avian covariates, we cross-referenced US occurrences of Order Passeriformes (perching birds) documented in the Cornell Lab of Ornithology eBird database [73] with those species analyzed by Jetz et al. [74] and archived in the Global Phylogeny of Birds (https://birdtree.org/) (accessed on 2 July 2021). We further cross-referenced the species common to both eBird (US occurrences only) and the Jetz et al. [74] phylogeny to the avian host competency database published by Tolsá et al. [54]. The Tolsá et al. [54] database reported estimated WNV host competency (molecular prevalence) for approximately half (163) of the 303 Passeriform species available from eBird and also represented in the global bird phylogeny.

We cloned the entire eBird database to the USDA SCINet High-Performance Computing System (https://scinet.usda.gov/) (accessed on 15 July 2021), extracted individual bird occurrences using the auk package [75], and then spatially and temporally matched occurrences by US County to produce a database indicating the presence or absence of each species in each county (3109) during each month of the year (January–December). To qualify as a presence, we required that a minimum of two species-specific observations be documented by eBird for a given county and month. We next constructed avian community matrices and calculated county-level species richness (number of unique species) under two different species pool assumptions: a “dynamic” species pool and a “static” species pool assumption. We use the term “dynamic” to refer to a species pool that varies intra-annually (monthly) such that the pool includes only those avian species present during a given month. By comparison, we use the term “static” to describe a species pool inclusive of all avian species observed during the combined months May–August, which define the primary WND outbreak season in the US [76,77].

Eco-phylogenetic analysis was conducted by first extracting 1000 bootstrap replicate trees from the Global Phylogeny of Birds using tools available at the https://birdtree.org/ (accessed on 12 July 2021) website. The tool facilitated the process of trimming the full, time-calibrated phylogeny to our Passeriform species pool (303 species) before sampling this subset from a pseudo−posterior distribution [74]. We then downloaded and summarized the replicate trees through construction of a maximum clade credibility tree using TreeAnnotator (http://beast.community/treeannotator) (accessed on 12 July 2021) and the BEAST 2 package [78]. We used the resulting consensus tree, our eBird based avian community matrices, and the picante package [79] to calculate mean phylogenetic distance (branch lengths between species), mean nearest taxon distance, which describes the average genetic distance between nearest neighbors (sister species) within a community [57], mean pairwise distance (average phylogenetic distance among co-occurring species pairs in a community, see Cadotte and Davies [80]), and evolutionary distinctiveness or the degree of a species’ isolation on the phylogeny [81]. As previously described for species richness, each phylodiversity metric was calculated under two different species pool assumptions: a monthly varying dynamic species pool and a static species pool representing the WND outbreak season of May–August. Each phylodiversity metric was compared to a null model derived from 999 random permutations of consensus tree tips and nationwide species pools (static and dynamic versions) to determine statistical importance and the degree of deviation from species pool averages.

Dynamic and static versions of avian host WNV molecular prevalence were estimated by matching species-specific prevalence estimates [54] to the corresponding bird species occurring in each county by month (dynamic) and during the peak outbreak season (static) before averaging across species (county-level mean community prevalence). Because avian host community composition varies through time (e.g., due to migration), averaging dynamic and static molecular prevalence by county produced estimates for mean host community prevalence that fluctuated intra-annually.

### 2.5. Land Cover and Human Demographic Information

To characterize typical land use and elevation by county, we obtained remote sensing data (GeoTiff format) from the Global 1 km Consensus Land Cover data set [82] and the Earth Environment Digital Elevation Model [83]. The land cover data set indicated the proportion of twelve different land cover types at a 1 km${}^{2}$ resolution. We aggregated elevation and land cover information to the county-level based on the mean elevation and the mean land cover proportion in each county. Descriptors for each land cover type are provided in the Results section and can be reviewed at the Earth Environment website http://www.earthenv.org/ (accessed on 15 July 2021).

County-level data reflecting human population density, median household income, and the percent of the population in poverty were obtained from the US Census Bureau (https://www.census.gov/) (accessed on 10 July 2021)and the Small Area Income and Poverty Estimates (SAIPE) Program within the US Census Bureau [84] using the censusapi package [85].

All data were scaled and centered for ease of post-modeling interpretation. Multicollinearity between candidate covariates was assessed using collinearity diagnostics for independent variables [86] as facilitated by the perturb package [87]. High multicollinearity between several covariates required that multiple model versions be constructed and evaluated. This iterative process is described further below.

### 2.6. Disease Model

Bayesian epidemiological models were constructed to estimate WND relative risk and disease caseloads for horses located in the conterminous US. Our statistical model was of the form,

(2) $$\begin{array}{cc} O_{\mathrm{st}}|r_{\mathrm{st}} & ∼\mathrm{Poisson}(\mu_{\mathrm{st}}) \end{array}$$

(3) $$\begin{array}{cc} \mu_{\mathrm{st}} & =E_{\mathrm{st}}r_{\mathrm{st}} \end{array}$$

(4) $$\begin{array}{cc} log(\mu_{\mathrm{st}}) & =log\left(E_{\mathrm{st}}\right)+log\left(r_{\mathrm{st}}\right), \end{array}$$

where the number of horse WND cases ($O_{\mathrm{st}}$) was conditional on relative risk ($r_{\mathrm{st}}$) and followed a Poisson distribution with a mean $\mu_{\mathrm{st}}$ (Equation (2)). $E_{\mathrm{st}}$ is the expected disease case counts in each US County *s* (s=1,2,3,…,3109) during each month *t* (t=January,February,March,…,December) between the years 2009 and 2017. Veterinarian reported WNV horse infections were obtained from the CDC as non-negative integers without any accompanying information describing the age, physical condition, or ownership of horses. Standardization by specific risk groups is the preferred method to estimate expected disease cases [88]. However, lacking detailed horse information, we calculated expected counts ($E_{\mathrm{st}}$) by multiplying the average rate for the period of record by the number of horses in each county (*s*).

Rearranging Equation (4) allowed for estimation of log risk using a number of random (non-linear) spatial and temporal effects as well as several fixed (linear) covariates of interest as potential risk indicators. The log-risk linear predictor can be represented as,

(5) $$\begin{array}{cc} log(r_{\mathrm{st}}) & =\alpha +\beta_{x}\cdot X_{st}+\zeta^{SVC}+\xi_{s}+\varphi_{t}+\gamma_{t}+\delta_{\mathrm{st}} \end{array}$$

(6) $$\begin{array}{cc} \zeta^{SVC} & =\sum_{k}^{m}f\;\left(\xi_{s}^{k}\;SVC_{st}^{k}\right) \end{array}$$

where α is an intercept approximating mean WND risk and the β $\left(\beta =\beta_{1},\ldots ,\beta_{x}\right)$ terms signify coefficients implemented as fixed covariates ($X_{st}$). The $\zeta^{SVC}$ shown in Equation (5) stands for spatially varying coefficients (SVC) and is detailed in Equation (6), where the *f*(·) represents statistical functions included to assess temperature and precipitation as spatially variable disease indicators. Because relationships between WND and climate were assumed to vary by location, climate covariates were designed to provide location-specific coefficient estimates. That is, rather than estimating a single coefficient that reflects the association of WND to temperature nationwide, the model instead provided a separate, “local” coefficient for WND–temperature correspondence in each US County. The $SVC_{st}^{k}$ term in *f*(·) represents temperature (k=1) or precipitation (k=2) at location *s* and time *t* with local coefficients that vary according to the latent Gaussian spatial process $\xi_{s}^{k}$. The spatial effect $\xi_{s}^{k}$ utilized a Besag formulation (Besag et al., 1991) to approximate a Gaussian Markov random field with individual counties considered conditionally independent unless adjoining as neighbors (sharing at least one connecting point along geographic boundaries).

In addition to the spatial covariates used to estimate SVCs, a separate spatial effect ($\xi_{s}$) was included to quantify model latencies (errors) due to unmeasured or unmodeled variables, spatial autocorrelation, and other data biases. The statistical implementation for $\xi_{s}$ was comparable to that of $\xi_{s}^{k}$ but incorporated a BYM configuration (Besag et al., 1991) with scaling between components [66,67,68,69] as described for WNV surveillance in Section 2.2. A zero mean constraint (centering on zero) was also enforced in $\xi_{s}$ to help reduce confounding between covariates. A zero mean constraint was not used for $\xi_{s}^{k}$ because doing so might have unintentionally altered the magnitudes of SVC estimates.

Beyond the spatial covariates described above, the model included spatiotemporal effects to account for ordered time ($\gamma_{t}$), unstructured time ($\varphi_{t}$), and space–time interaction ($\delta_{\mathrm{st}}$). Ordered time ($\gamma_{t}$, Equation (5)) was specified using a first-order random walk as used in Section 2.2 to temporally smooth virus detection estimates. Unstructured time ($\varphi_{t}$, Equation (5)) and space–time interaction ($\delta_{\mathrm{st}}$, Equation (5)) were modeled as independent and identically distributed random effects with months (time steps) used as variable levels for unstructured time and county-month combinations used as variable groups for space–time interaction. The random walk helped identify within year time trends, the unstructured time effect captured temporal variation outside of the ordered time trend, and space–time interaction helped detect locations that departed from average risk trends for the study period. Because our model exhibited high-dimensionality, we applied Integrated Laplace Approximation using the INLA package as an alternative to computationally demanding Markov chain Monte Carlo methods [89,90,91]. Spatiotemporal effects were specified with weakly informative Penalizing Complexity priors [69,92] having parameters $p_{1}=1$ and $p_{2}=0.001$ with enforced zero mean constraints to help reduce confounding. All fixed effects were assigned vague zero mean normal priors with a 0.0001 precision.

### 2.7. Model Selection, Consensus, and Validation

**Figure A1:**
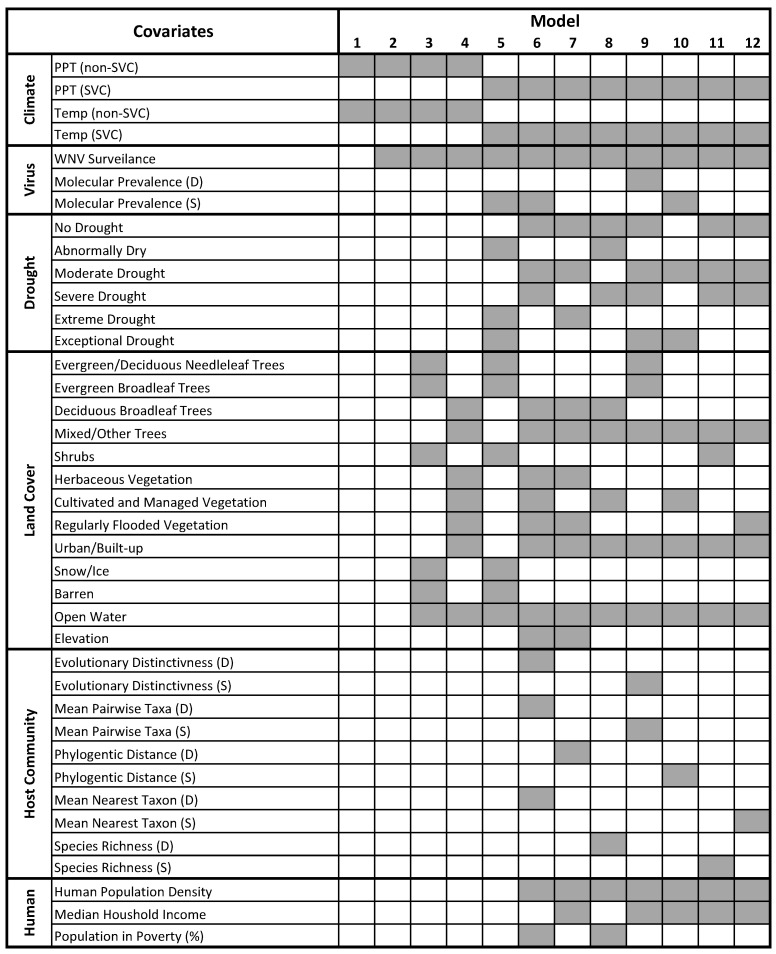
Evaluated model covariates. Covariates are listed by category in the first column. Columns following covariate name correspond to twelve models constructed to evaluate covariates (numbered 1–12) and are shaded by row to indicate covariate inclusion. The abbreviation SVC when listed with a covariate name indicates implementation as a Spatially Varying Coefficient, parenthetical listing of (D) signifies the covariate was added under assumption of a dynamic species pool (monthly varying), and parenthetical (S) indicates use of a static pool (seasonal, months May–August). Models 5–12 included climate SVC covariates and were subjected to model averaging to derive mean consensus estimates of WND risk.

**Figure A2:**
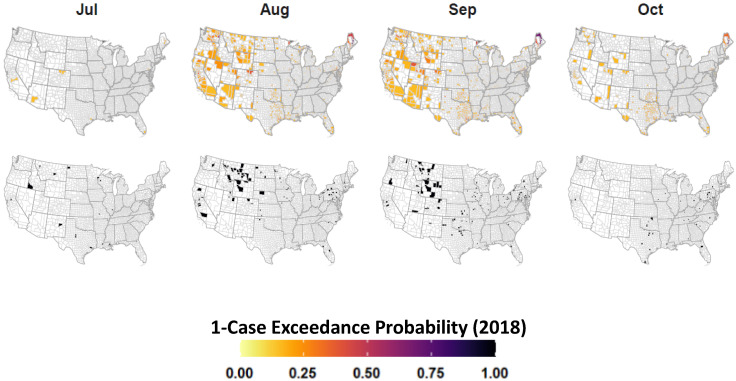
Model validation. Top row depicts out-of-sample, model predicted 1-case exceedance probability for the months July, August, September, and October 2018. Bottom row depicts locations of actual 1-case exceedance as reported to the CDC.

A total of 39 climatic, phylogenetic, and environmental variables were assessed as potential equine WND risk indicators. To avoid multicolinearity among variables, a consensus modeling approach was adopted such that 12 different models were iteratively constructed using data years 2009 to 2017 before application of model averaging [93,94]. As previously described, collinearity diagnostics for independent variables [86] were applied to ensure that the variable combinations specific to individual models posed a low risk of multicolinearity. Comparison of the 12 candidate models revealed that marginal likelihoods and Watanabe-Akaike information criteria (WAIC) among the top 7 models fell within 1% of each other. Therefore, each model was assigned even weighting during model averaging (mean consensus). Because correlative relationships among input covariates and between the covariates and estimated risk were potentially informative from a systems perspective, we visualized all correlative relationships concurrently through network analysis [95,96]. Model validation was conducted through comparison of averaged model estimates to cases reported in 2018 (out-of-sample) using Brier [97] and logarithmic [98] scores. To accomplish this, model predicted 1-case exceedance probabilities for equine WND cases were compared to the county and month-specific case counts reported in 2018. Network analyses conducted during initial covariate selection and model development were then repeated following model validation to concurrently assess relationships between estimated WND risk and the original input covariates. A list of covariates specific to each model and maps depicting 1-case exceedance probabilities in relation to 2018 reported cases are provided in Appendix A (see Figure A1 and Figure A2).

## 3. Results

**Figure 1:**
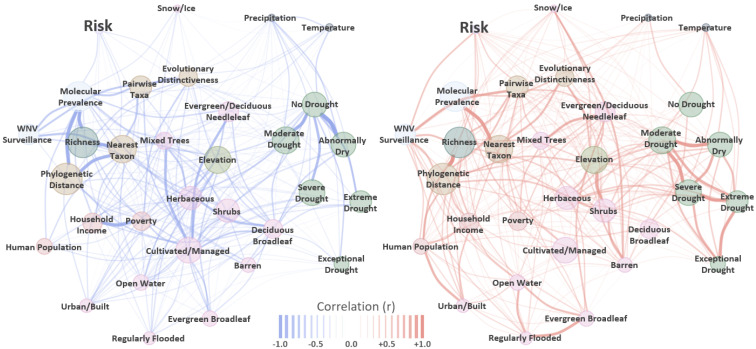
Network—correlation correlation graph. Networks display negative (**left**) and positive (**right**) correlations among evaluated input covariates and model estimated risk (larger text, upper left in each graph). Network nodes are labeled to indicate model covariate name and are sized to reflect the absolute magnitude of average Pearson linear correlation (r). Graph edges (lines) are color coded to indicate polarity (blue = negative, red = positive) with widths sized according to legend at bottom to signify absolute magnitude of pairwise correlation (range = −1 to +1). Graph structure among node positions (groups, clusters, or nestedness) approximate average connectivity (“node comunities”).

Network analysis revealed graph structure (network graph topology) among disease risk indicators (graph nodes) such that covariates from similar groups (e.g., climate, host phylogenetics, land cover) were positioned in relatively close proximity whereas covariates from different groups were at distance (Figure 1). For example, avian species richness (Richness) exhibited strong negative correlation to virus molecular prevalence (Figure 1 [left graph]) and a robust positive correlation to phylogenetic distance (Figure 1 [right graph]), yet these three variables were clustered (grouped) together with other avian community covariates (e.g., nearest taxon, pairwise taxa) due to overall similarity. In a comparable manner, covariates reflecting different drought stages were clustered (Figure 1, right side of both graphs) as were those for land cover (Figure 1, bottom center of both graphs). Estimated risk occupied a graph position nearest virus covariates (prevalence and surveillance) and avian host factors suggesting stronger correlative relationships to these indicators than to land cover or climate variables.

**Figure 2:**
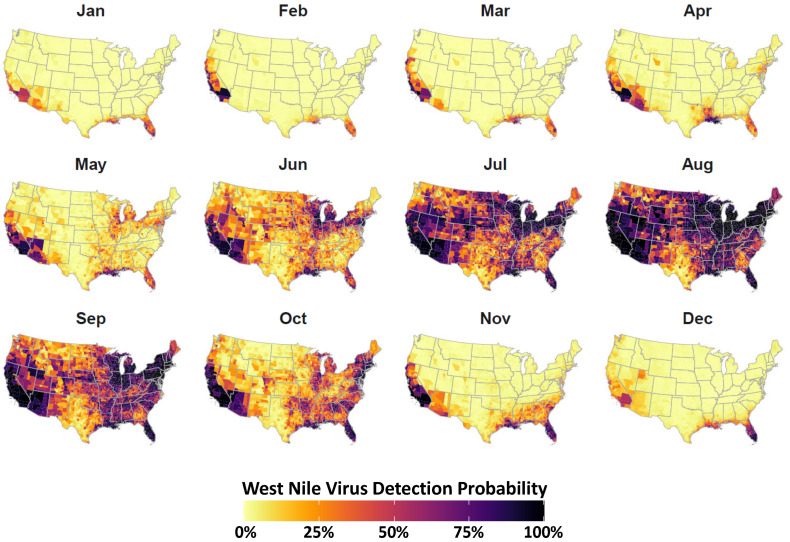
WNV detection probability. WNV surveillance covariate estimated from virus detections reported to the CDC. Mapped counties are color coded according to legend at bottom to indicate WNV detection probability (converted to percent chance). Darker tones indicate an elevated chance of virus detection whereas lighter tones represent a lesser chance of detection. Covariate construction is detailed in Section 2.2.

Spatial and temporal smoothing of reported WNV detections from sentinel animal, bird, and mosquito surveillance produced monthly, county-specific estimates for WNV detection probability in the US (Figure 2). The WNV surveillance covariate was found to be an important predictor of WND risk with increased virus detection probability corresponding to increased disease risk (Table 1).

**Figure 3:**
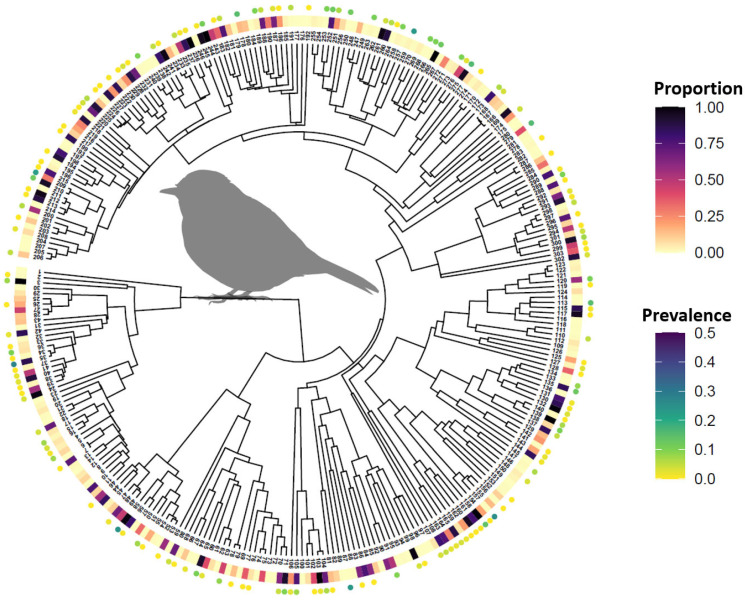
Phylogenetic tree for WNV avian hosts. Phylogenetic tree for 303 Passeriform species. Rectangular boxes near tree tips are color coded according to the legend at top right (Proportion) to indicate the proportion of US Counties where each species has been documented to occur. Rectangles coded as dark red indicate the species occurs in a high proportion of counties whereas lighter, yellow rectangles indicate relatively lower proportions. Circles surrounding tree tips correspond to legend at bottom right (Prevalence) and signify species-specific WNV molecular prevalence. Tree tips without circles indicate that prevalence information was not available at time of analysis. Figure A3 provided with Appendix A lists species names, proportion of occupied counties, and prevalence values for each tree tip.

**Figure A3:**
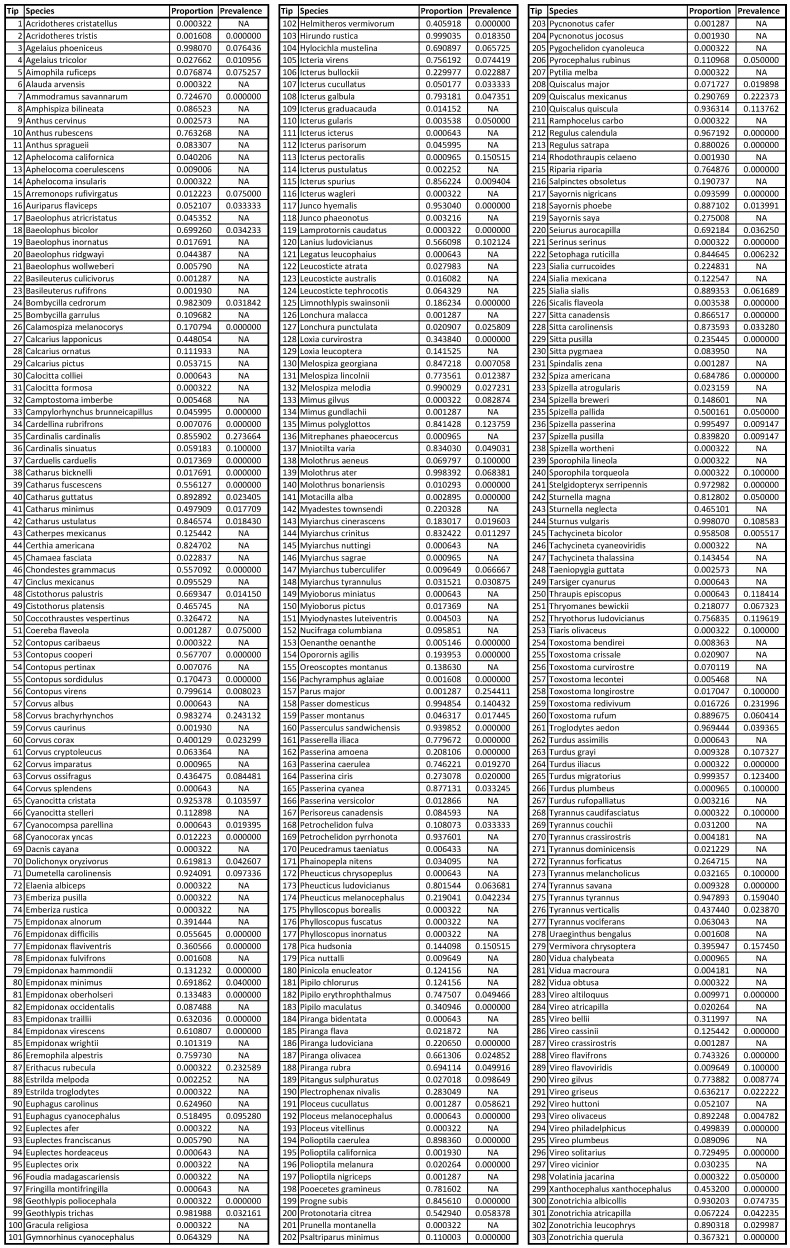
Detailed metadata for WNV avian host phylogenetic tree (Figure 3). Columns list the tree tip number (Tip), species name (Species), the proportion of occupied counties (Proportion), and estimated WNV prevalence for each avian species.

We identified 303 Passeriform species common to both the Cornell Lab of Ornithology eBird database [73] and the Global Phylogeny of Birds [74]. Metadata symbolized with the maximum clade credibility tree show the proportion of US Counties where each species has been observed and documented by the eBird database (Figure 3). The tree also indicates the species-specific WNV molecular prevalence as estimated by Tolsá et al. [54]. Figure A3 provided with Appendix A lists species names, the proportion of occupied counties, and prevalence for each tree tip.

**Figure 4:**
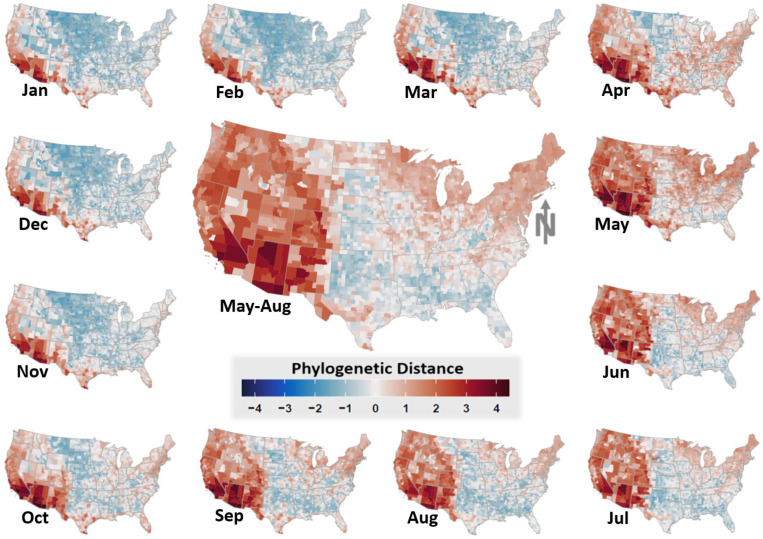
Dynamic and static avian phylogenetic distance. Phylogenetic distance covariate under assumption of dynamic (monthly) and static (seasonal) species pools. Dynamic version is shown as a monthly varying time-series surrounding the larger map at center, which represents static phylogenetic distance (May–August). Mapped values have been scaled and centered to highlight locations subject to relative phylogenetic clustering with blue colors (higher than expected relatedness, lower mean genetic distances) and phylogenetic over-dispersion with red colors (lower than expected relatedness, higher mean genetic distances).

**Figure A4:**
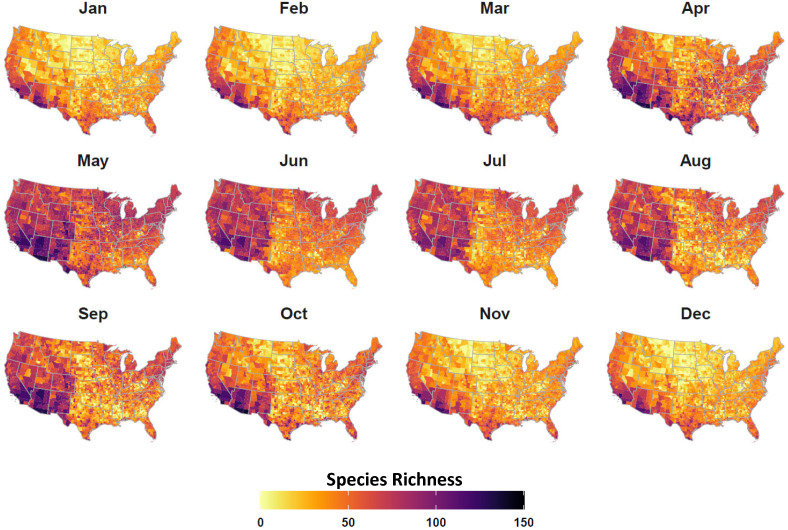
Dynamic avian host species richness. Species richness covariate under assumption of a dynamic species pool (monthly varying). Maps are color coded according to legend at bottom with darker tones indicating higher species richness.

**Figure A5:**
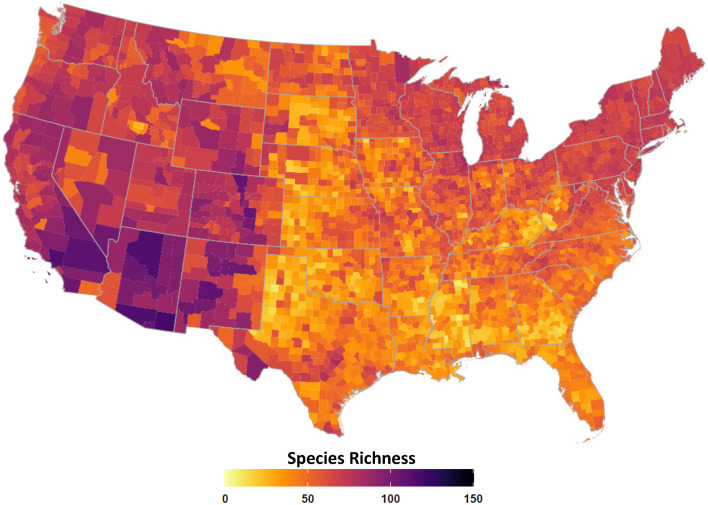
Static avian host species richness. Species richness covariate under assumption of a static species pool (May–August). Map is color coded according to legend at bottom with darker tones indicating higher species richness.

**Figure A6:**
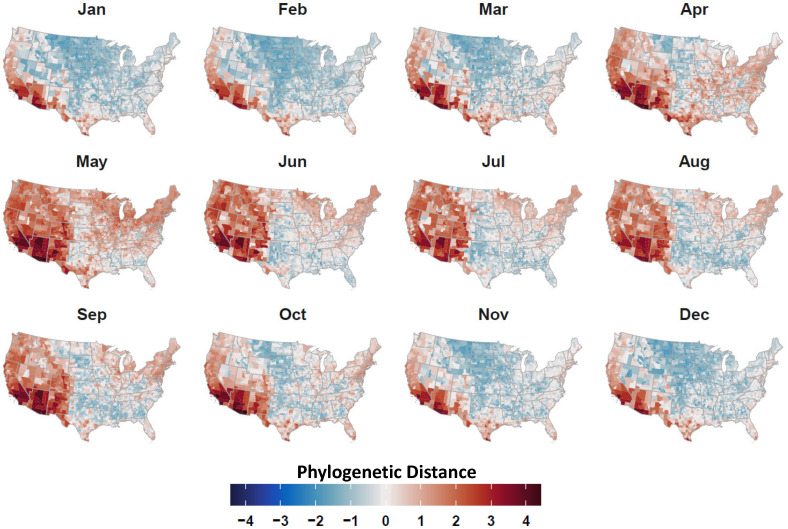
Dynamic avian phylogenetic distance. Phylogenetic distance covariate (sum of branch lengths) under assumption of a dynamic species pool (monthly varying). Mapped values have been centered to highlight locations subject to relative phylogenetic clustering (higher than expected relatedness, blue colors) and dispersion (lower than expected relatedness, red colors).

**Figure A7:**
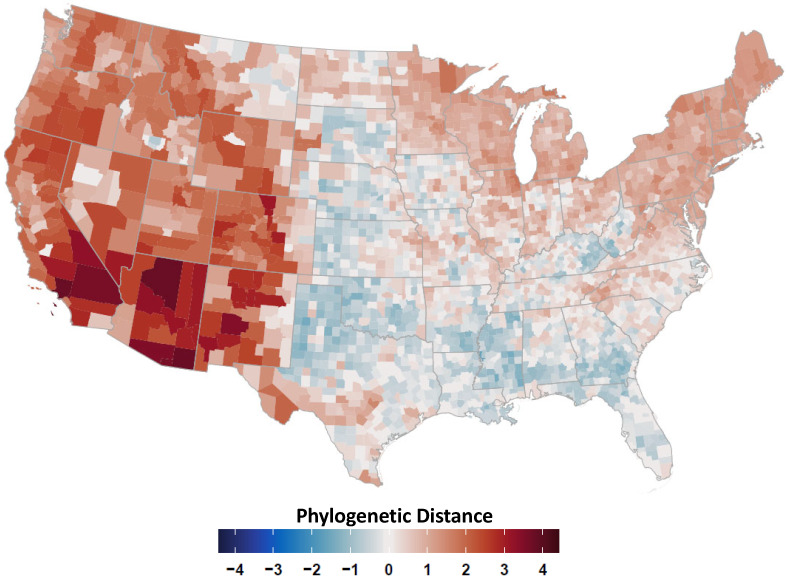
Static avian phylogenetic distance. Phylogenetic distance covariate (sum of branch lengths) under assumption of a static species pool (May–August). Mapped values have been centered to highlight locations subject to relative phylogenetic clustering (higher than expected relatedness, blue colors) and dispersion (lower than expected relatedness, red colors).

**Figure A8:**
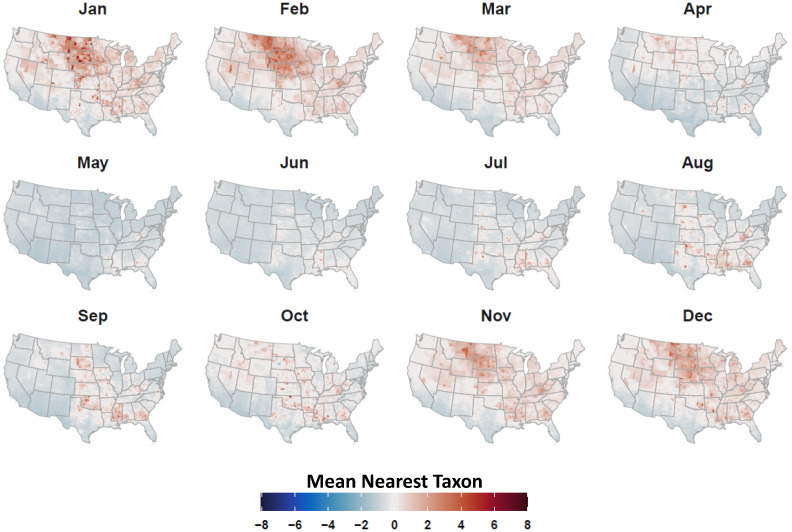
Dynamic avian mean nearest taxon distance. Mean nearest taxon covariate under assumption of a dynamic species pool (monthly varying). Covariate reflects average genetic distance between nearest neighbors (sister species) within a community. Mapped values have been centered to highlight locations subject to relative phylogenetic clustering (higher than expected relatedness, blue colors) and dispersion (lower than expected relatedness, red colors).

**Figure A9:**
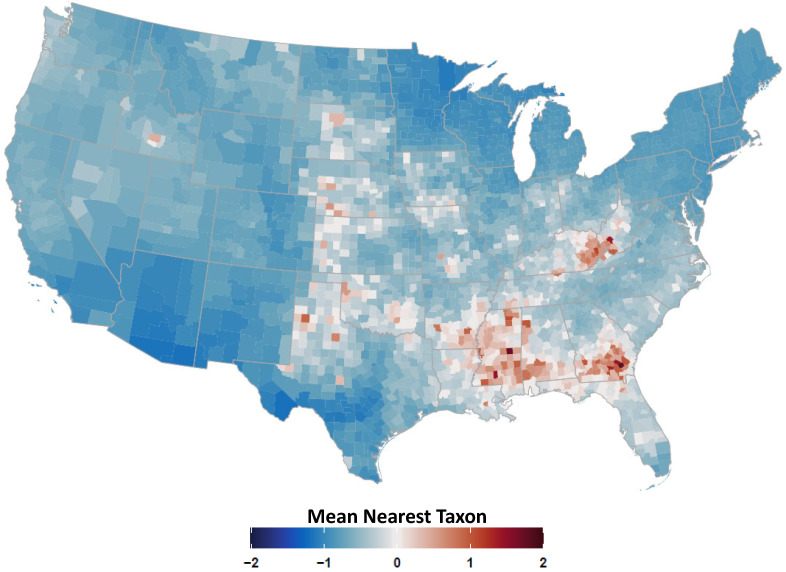
Static avian mean nearest taxon distance. Mean nearest taxon covariate under assumption of a static species pool (May–August). Covariate reflects average genetic distance between nearest neighbors (sister species) within a community. Mapped values have been centered to highlight locations subject to relative phylogenetic clustering (higher than expected relatedness, blue colors) and dispersion (lower than expected relatedness, red colors).

**Figure A10:**
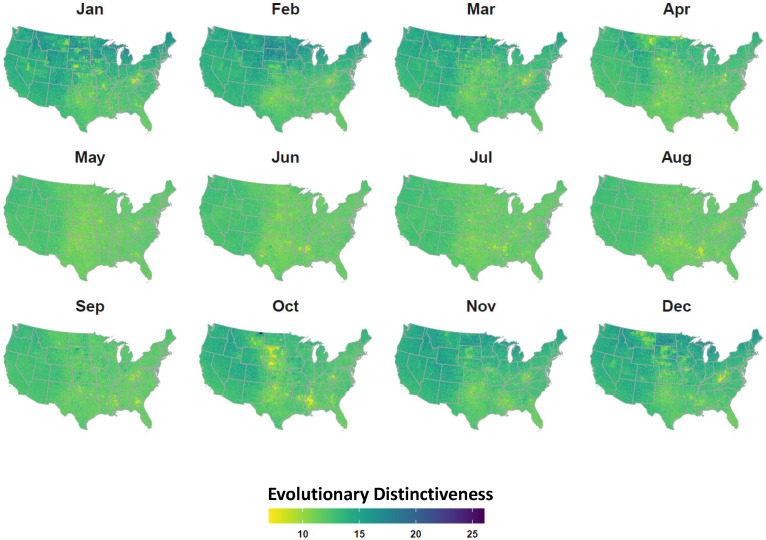
Dynamic avian evolutionary distinctiveness. Evolutionary distinctiveness covariate under assumption of a dynamic species pool (monthly varying). Covariate reflects degree of species isolation on the phylogeny or the average distance of a species to all other species in the community. Mapped counties are color coded according to legend at bottom to indicate increased evolutionary distinctiveness with darker tones.

**Figure A11:**
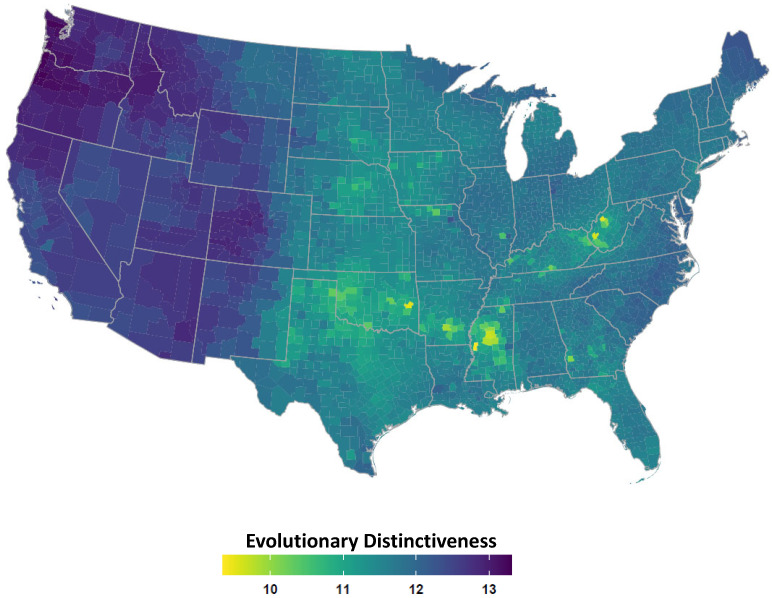
Static avian evolutionary distinctiveness. Evolutionary distinctiveness covariate under assumption of a static species pool (May–August). Covariate reflects degree of species isolation on the phylogeny or the average distance of a species to all other species in the community. Mapped counties are color coded according to legend at bottom to indicate increased evolutionary distinctiveness with darker tones.

**Figure A12:**
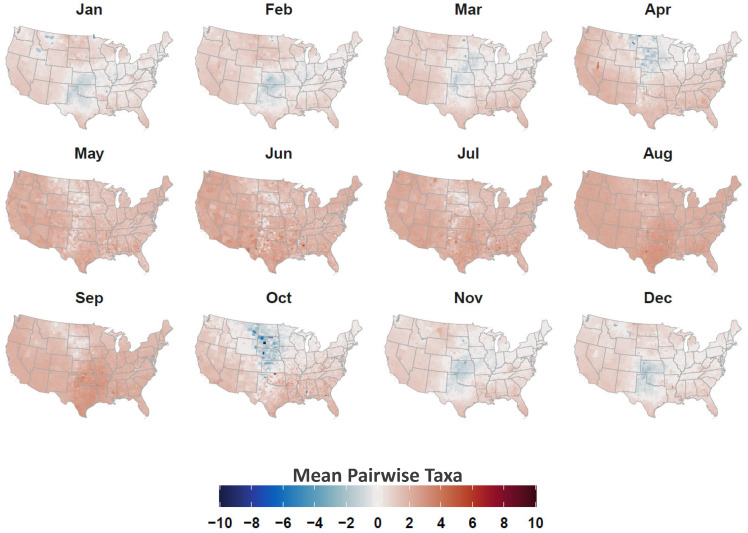
Dynamic avian mean pairwise taxa distance. Mean pairwise taxa covariate under assumption of a dynamic species pool (monthly varying). Covariate reflects average phylogenetic distance among co-occurring species pairs in a community. Mapped values have been centered to highlight locations subject to relative phylogenetic clustering (higher than expected relatedness, blue colors) and dispersion (lower than expected relatedness, red colors).

**Figure A13:**
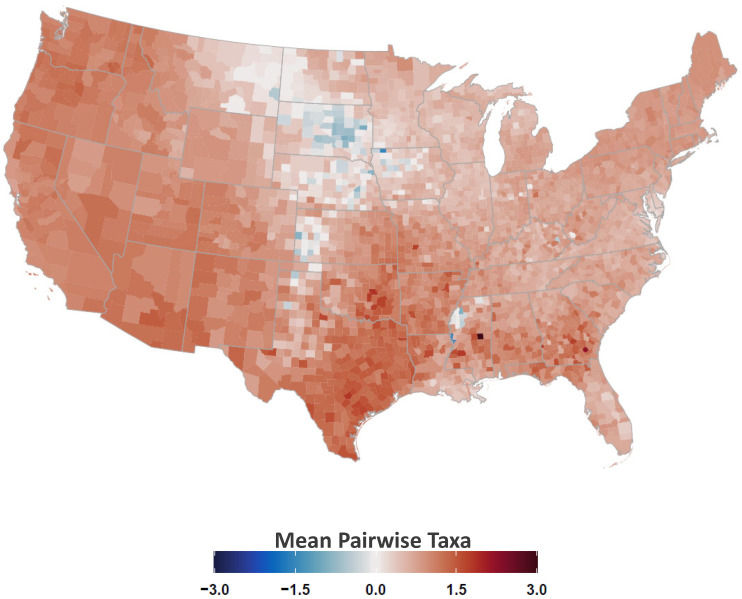
Static avian mean pairwise taxa distance. Mean pairwise taxa covariate under assumption of a static species pool (May–August). Covariate reflects average phylogenetic distance among co-occurring species pairs in a community. Mapped values have been centered to highlight locations subject to relative phylogenetic clustering (higher than expected relatedness, blue colors) and dispersion (lower than expected relatedness, red colors).

**Figure A14:**
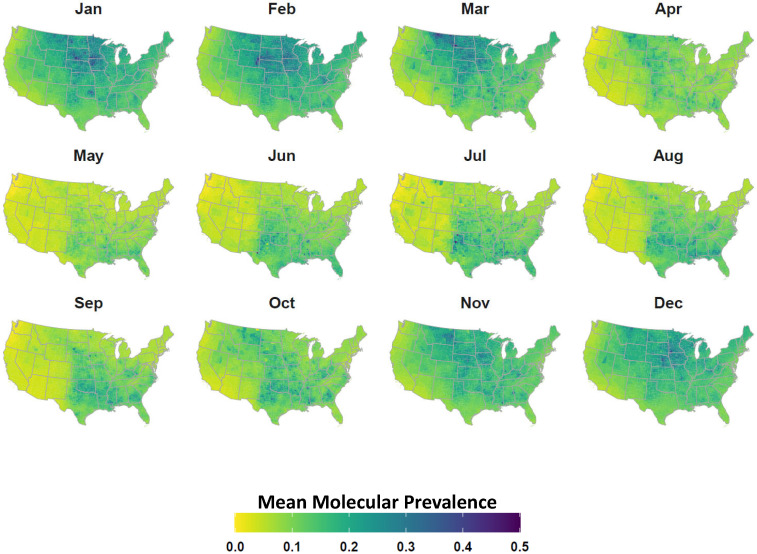
Dynamic avian WNV mean molecular prevalence. Molecular prevalence covariate under assumption of a dynamic species pool (monthly varying). Covariate reflects avian community average WNV prevalence based on estimates reported by Tolsá et al. [54]. Mapped counties are color coded according to legend at bottom to indicate increased WNV prevalence with darker tones.

**Figure A15:**
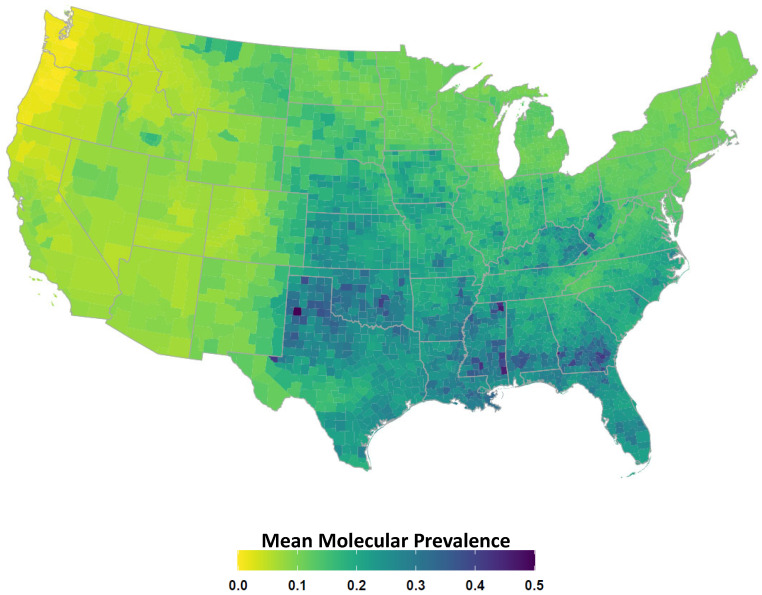
Static avian WNV mean molecular prevalence. Molecular prevalence covariate under assumption of a static species pool (May–August). Covariate reflects avian community average WNV prevalence based on estimates reported by Tolsá et al. [54]. Mapped counties are color coded according to legend at bottom to indicate increased WNV prevalence with darker tones.

Combining avian host phylogenetics (Figure 3) with bird occurrence and community composition information allowed for the estimation and mapping of several phylodiversity metrics and average host community WNV prevalence. Figure 4 illustrates the phylogenetic distance metric as a representative example of these results. However, outcomes for all phylodiversity and prevalence metrics are provided in Appendix A as maps (see, Figure A4, Figure A5, Figure A6, Figure A7, Figure A8, Figure A9, Figure A10, Figure A11, Figure A12, Figure A13, Figure A14, Figure A15). As exemplified by phylogenetic distance (Figure 4), estimating community phylogenetic composition from a temporally dynamic perspective indicated that average relatedness varied considerably throughout the year.

**Figure 5:**
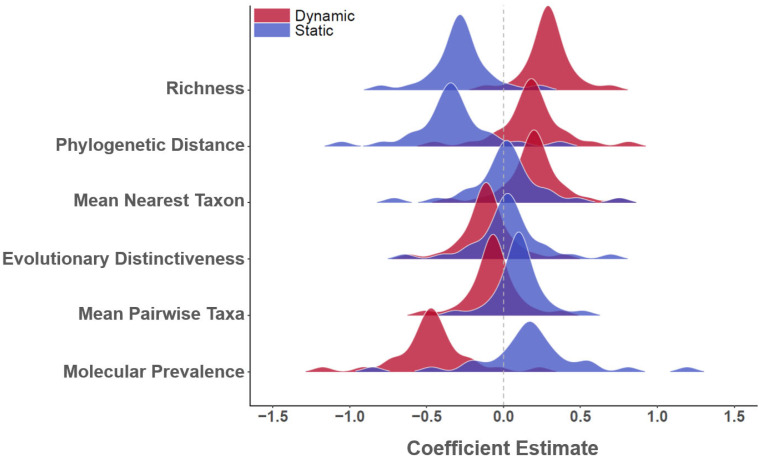
Avian host community composition, phylodiversity, and WNV prevalence posterior distributions. Vertical axis at left lists covariate names and horizontal axis provides numeric range of coefficient estimates. Distributions are color coded to indicate if the estimate corresponds to a dynamic (monthly varying) or static (season-based, May–August) avian species pool. Dashed vertical line intersects zero on horizontal axis to judge credible intervals and covariate polarity. The static implementations of mean nearest taxon distance, evolutionary distinctiveness, and molecular prevalence were determined not to be statistically significant based on 95% credible intervals. Neither the static or dynamic versions of mean nearest taxa distance were significant. All other other covariates were important indicators of WND risk.

The strength and importance of avian host community metrics in estimating WND risk differed by adopted species pool and the specific composition measure (Figure 5). Coefficient estimates for species richness, phylogenetic distance, mean nearest taxon distance, evolutionary distinctiveness, mean pairwise taxa distance, and molecular prevalence are shown under both the dynamic and static species pool assumptions. Coefficients estimated under the dynamic and static assumptions exhibited contrasting polarity (positive or negative signs) within the same covariate and showed differing influence with respect to predictive power. The static implementations of mean nearest taxon distance, evolutionary distinctiveness, and molecular prevalence were determined not to be statistically significant based on 95% credible intervals, nor were either the static or dynamic versions of mean nearest taxa distance. All other covariates were found to be important indicators of WND risk (Figure 5).

**Figure 6:**
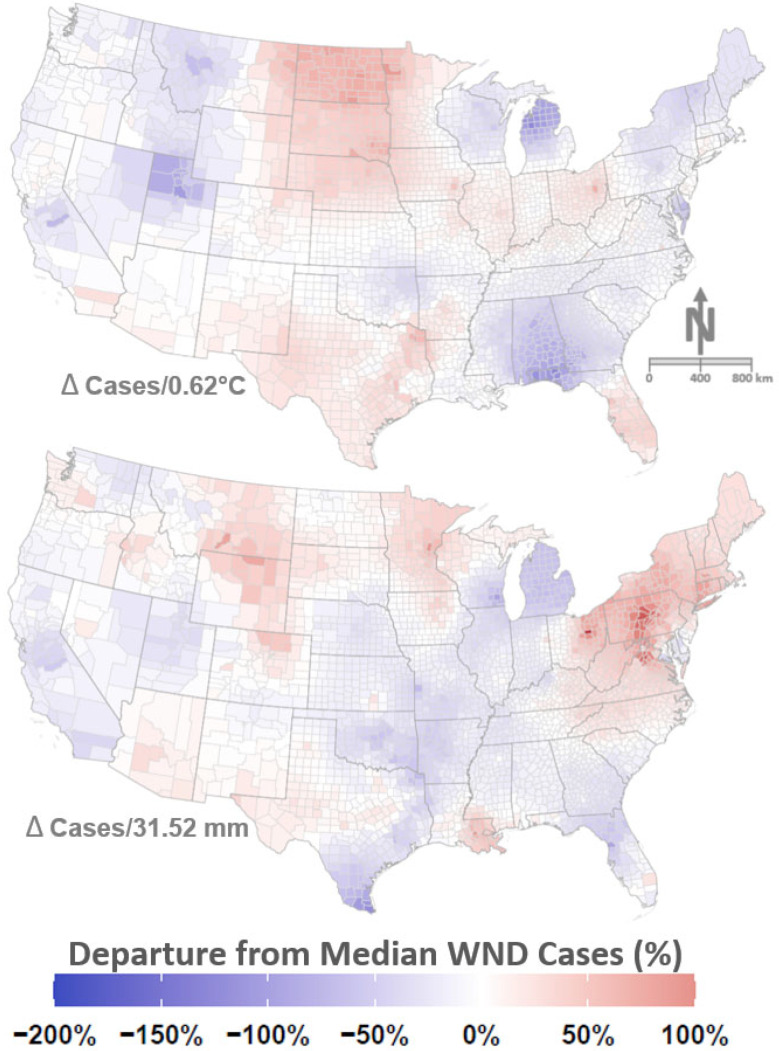
Spatially varying coefficients (SVCs) for climate. Maps show SVCs for temperature (**top**) and precipitation (**bottom**) by US County. Mapped colors correspond to legend at bottom and are scaled to show relative change (%) in equine WND cases with respect to the median case rate of 3.88 (1.83, 6.67 CI) cases/100,000 horses. Warm colors (reds) highlight locations where above average temperature (per 0.62 ${}^{{}^{\circ}}$C anomaly) and precipitation (per 31.52 mm anomaly) correlate to increased WND cases. Cooler colors (blues) indicate locations where above average temperature and precipitation correlate with decreased WND cases. Areas shown in white signify locations with little change in WND cases as temperature or precipitation increase.

Initial inclusion of temperature and precipitation climate variables as fixed model covariates (Models 1–4, see Figure A1) indicated that both covariates were not significant as judged by 95% Credible Intervals including the value 0 (zero). However, temperature and precipitation were found to be important when added as spatially varying coefficients (Models 5–12, see Figure A1). The relative influence (“effect sizes”) of temperature and precipitation covariates varied by location and are mapped by US County (Figure 6).

**Figure 7:**
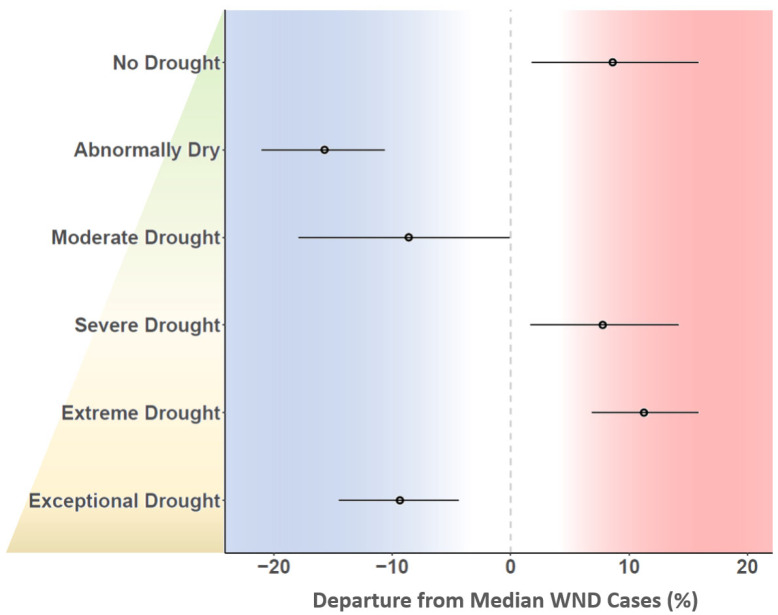
Relationship of drought to equine WND. Vertical axis at left lists US Drought Monitor categories arranged (**top** to **bottom**) from the least to most dry stage. Horizontal axis is scaled to show relative change (%) in equine WND cases with respect to the median case rate of 3.88 (1.83, 6.67 CI) cases/100,000 horses, which is represented by the dashed vertical line intersecting zero. Point symbols in main plot area represent the mean coefficient estimate for each drought category with a corresponding line defining the 95% CI.

Drought indices were found to be statistically significant in estimating WND risk and produced coefficients with polarity that differed by drought category and intensity (Figure 7). The number of WND cases increased as the proportion of land classified as No Drought (0), Severe Drought (3), and Extreme Drought (4) increased. Conversely, WND cases decreased as land proportions in the Abnormally Dry (1), Moderate Drought (2), and Exceptional Drought (5) categories increased, indicating that drought thresholds are important in understanding WND risk.

WNV surveillance, median household income, and four land cover types were statistically important indicators of WND risk (Table 1). WNV surveillance and the proportion of Barren land cover in a county exhibited positive correlation to increased WND cases whereas other significant variables showed a negative correspondence to WND. Among covariates negatively associated with WND was median household income. Coefficients estimated for median household income indicated that as average income increased within a county, risk of WND in horses proportionality decreased.

**Figure 8:**
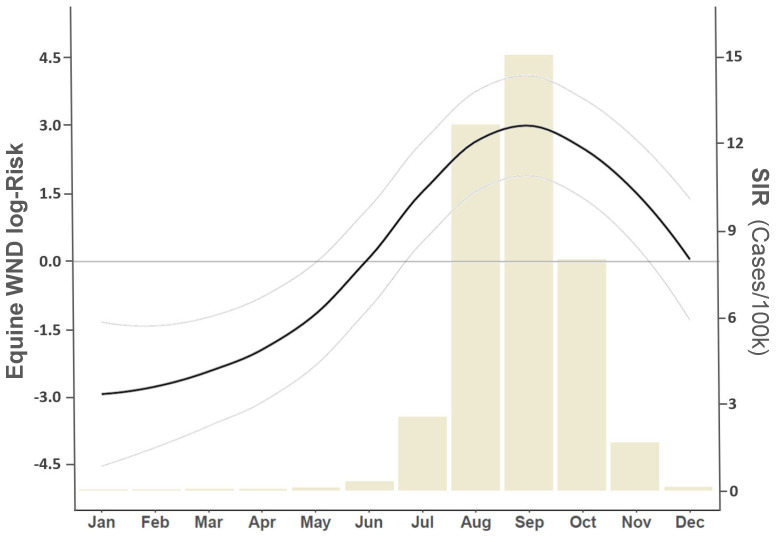
Temporal distribution of equine WND relative risk. Vertical axis at left describes model estimated log-risk (absolute risk, case counts on the log scale) and corresponds to smooth curve reflecting intra-annual changes in case intensity. Light gray lines surrounding smooth curve demarcate the estimated 95% CI. Horizontal gray line intersecting 0 (zero) on the left vertical axis represents the US annual median case rate of 3.88 (1.83, 6.67 CI) cases/100,000 horses. Horizontal axis at bottom lists the month of year. Bar chart in background corresponds to right vertical axis providing monthly standardized incidence rates (SIR).

**Figure 9:**
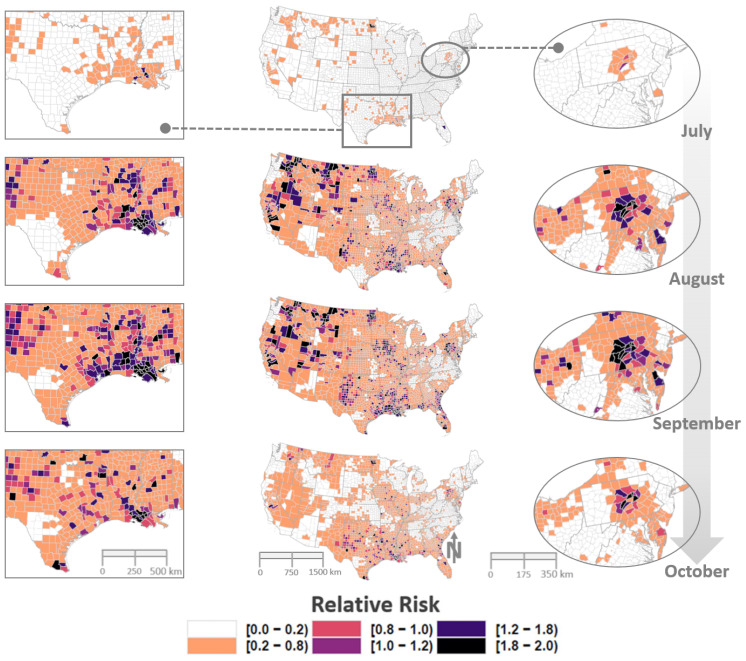
Spatiotemporal distribution of equine WND relative risk. Maps depict the spatial and temporal distribution of model estimated WND relative risk by US County for the months July–October. Column aligned at center displays the Contiguous US with lateral columns providing closer views of locations demarcated on US map at top center. Maps are color coded according to legend at bottom such that darker tones signify increased risk and lighter tones represent relatively lower risk. A relative risk value of 1 indicates that model predicted cases were comparable to the expectation given the number of horses in the county, values below 1 highlight counties with relatively low risk, and values above 1 suggest increased risk (higher than expected given the horse population).

**Figure A16:**
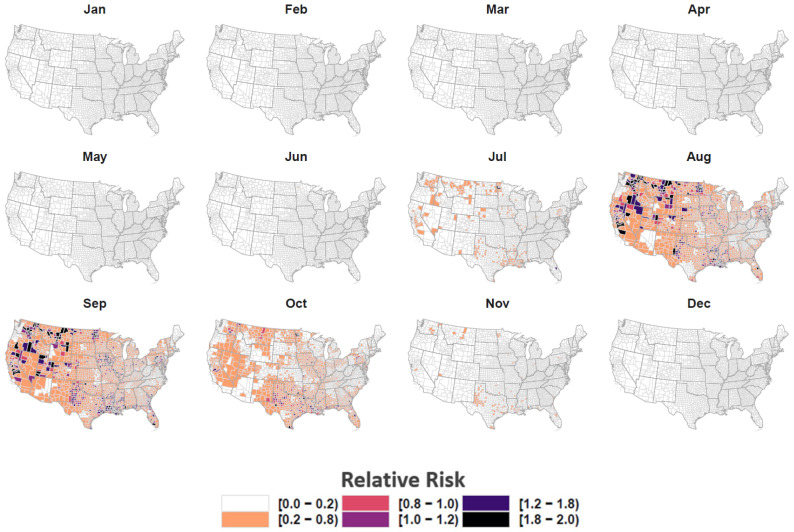
Spatiotemporal distribution of equine WND relative risk. Maps depict the spatial and temporal distribution of model estimated WND relative risk by US County and month of year. Maps are color coded according to legend at bottom such that darker tones signify increased risk and lighter tones represent relatively lower risk. A relative risk value of 1 indicates that model predicted cases were comparable to the expectation given the number of horses in the county, values below 1 highlight counties with relatively low risk, and values above 1 suggest increased risk (higher than expected given the horse population).

**Figure A17:**
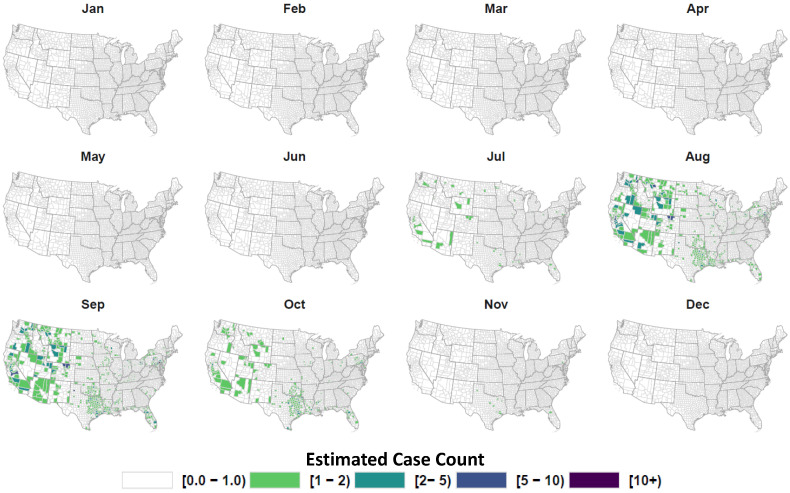
Estimated equine WND case counts. Maps depict the spatial and temporal distribution of model estimated WND cases by US County and month of year. Maps are color coded according to legend at bottom such that darker tones signify increased case loads and lighter tones represent relatively lower case counts.

**Figure A18:**
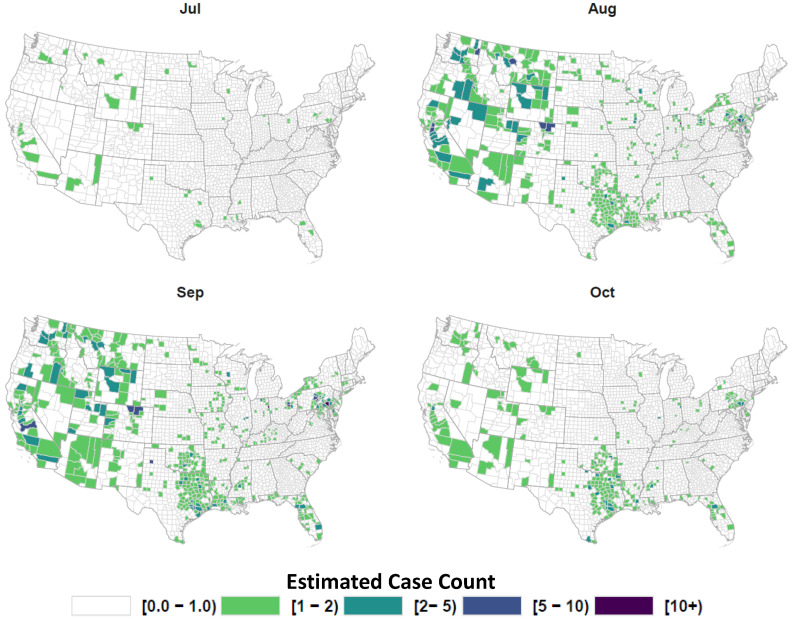
Estimated equine WND case counts. Maps depict the spatial and temporal distribution of model estimated WND cases by US County for select months during the peak WND outbreak season. Maps are color coded according to legend at bottom such that darker tones signify increased case loads and lighter tones represent relatively lower case counts. Case counts for all months of the year are provided with Appendix A (see Figure A17).

The annual median case rate for equine WND across all US Counties was approximately 3.88 (1.83, 6.67 CI) cases/100,000 horses. However, the distribution of WND risk exhibited a temporal trend that sharply increased between the months of June and August (Figure 8) and showed considerable spatial heterogeneity throughout the year (Figure 9 and Figure A16). Spatial and temporal trends aligned to indicate July–October as months of highest disease risk with time periods before and after showing markedly decreased disease rates (Figure 9 and Figure A16). The spatiotemporal distributions of estimated case counts were comparable to those shown for relative risk and are illustrated in Appendix A (see Figure A17 and Figure A18).

## 4. Discussion

As expected, equine WND risk was not uniformly distributed across the US nor was it constant throughout the year. Nationwide risk patterns generally indicated that few locations were free of any disease risk during the July–October period, which was identified as the time of highest risk (Figure 8). However, several multi-county regions exhibited particularly elevated risk (Figure 9) during this timeframe. High-risk clusters (relative risk ratio > 1.0) were identified in Central Pennsylvania, Eastern Iowa, West Texas, Central Montana, Coastal South Atlantic States, Northwest Minnesota, Eastern Washington, the Idaho-Oregon border, and along the central Gulf coast in a region centered on Lower Louisiana. Clusters in Central Pennsylvania and Lower Louisiana were the first high-risk, multi-county areas to emerge following the start of the outbreak season and remained as the most persistent risk areas as summer transitioned to the fall season. Interestingly, the Louisiana and Pennsylvania clusters underlie migratory flight paths linking the Gulf Coast to the Northeastern US that were previously identified as routes of WNV transport by terrestrial bird (non-waterfowl) species [45]. In addition to high-risk clusters, a number of relatively isolated areas (1–2 adjoining counties) showed disproportionately high relative risk based on the associated horse population. For example, individual counties in Colorado, North Dakota, and California displayed risk twice as high as expected (relative risk ratio ⩾2.0). Being a population contingent measure, the variability observed in risk distribution suggested that, although WNV has been detected throughout the US, environmental conditions at some times and locations are more conducive to disease propagation than are conditions at other times and locations.

Our analysis revealed that risk spatial and temporal heterogeneity was associated with a number of virus, avian host, and climatic factors. Perhaps the most intuitive indicator of disease was detection of the WNV itself, which was found to be the strongest risk correlate during network analysis (Figure 1) and a robust risk indicator through spatiotemporal modeling (Figure 2). When mapped as a time-series, virus detection probability suggested a general shift from south-to-north during the onset of summer (May–June), a majority coverage of the US during the height of summer (June–September), and a north-to-south recession associated with the beginning of winter (October–November). During the coldest months of year (November–April), highest virus occurrence probabilities were predominantly restricted to southern portions of the US and coastal areas. The apparent annual movement by WNV aligned with seasonal turnover in the US such that virus detection likelihood increased along a latitudinal gradient as temperatures warmed. Exceptions to this latitudinal pattern were identified along the Atlantic and Pacific Coasts where relatively high virus detection probabilities persisted throughout the winter months. We interpret these aberrations from the prevailing pattern to be linked to thermal buffering of coastal areas by oceans. Thermal buffering moderates low-temperature extremes in coastal areas, among other effects, to provide winter refuge for birds (potential reservoirs) that might otherwise migrate [99,100]. However, it is also possible that managed water and sewage systems in some coastal locations facilitate mosquito overwintering [101].

Considering the large geographic extent of our study area, temperature and precipitation were presumed to exhibit spatial non-stationarity with respect to disease risk and were modeled as spatially varying coefficients (SVC). Spatial non-stationarity describes ecological relationships that vary by geographic location or across spatial scales [102,103]. As an example of non-stationarity in a disease system, Olson et al. [104] found that precipitation was correlated to elevated malaria incidence in upland areas of the Amazon basin where water availability was a limiting factor for mosquito habitat. However, precipitation was negatively correlated with malaria in wetlands, where additional rainfall washed out existing mosquito habitats adequate for reproduction. Just as precipitation showed contrasting effects on malaria incidence for upland and wetland locations in the Amazon, we anticipated that precipitation’s influence on WND risk would vary by location due to flexible responses by WNV vectors [105] and underlying heterogeneity in prevailing environmental conditions throughout the US. For example, we assumed that increased rainfall would affect WNV enzootic transmission in arid and semi-arid locations such as the Sonoran and Chihuahuan deserts differently than it would in water abundant regions like those found in the Southeast.

Precipitation coefficients exhibited a spatial distribution (Figure 6) with increased precipitation corresponding to elevated WND risk across the northernmost western states, the Northeast, and the majority of Mid-Atlantic states. Increased precipitation was correlated with decreased disease risk in states laying immediately west of the Mississippi flow way, the Southeast, and California. Temperature has been previously linked to WND outbreaks in the US [106,107,108]. The resulting pattern shown for temperature coefficients (Figure 6) indicated that increasing temperature was correlated with increased WND risk across the Midwest, the Florida peninsula, Texas, and the Dakotas but negatively associated with WND in the Upper Midwest, non-coastal western states, the extreme Northeast, and the states of Mississippi, Alabama, and Georgia. Although the regional patterning shown by mapped temperature and precipitation results (Figure 6) differed with respect to each other, both largely tracked recognized US regional climatic boundaries [109].

Drought demonstrated a dynamic relationship to WND in that non-drought conditions were associated with increased risk, but abnormal dryness and moderate drought had a suppressive effect on disease (Figure 7). As drought intensity increased beyond moderate levels to reach the severe and extreme stages, disease risk sharply increased before once again diminishing to have a negative disease influence during periods of exceptional drought. The dynamic, non-linear correspondence between drought and WND as drought conditions intensified may be indicative of shifts in vector and host availability. Although moderate drought conditions may reduce host and vector water access, severe drought levels may exacerbate the situation sufficiently to instigate vector and host aggregation at the few remaining water sources. As drying conditions further intensify to be classified at the exceptional level, water becomes so rare as to be a limiting factor for virus transmission. Drought and other weather extremes may promote pathogen transmission by concentrating vector and host populations in relatively small areas [107,110,111]. Mosquito vector and avian host aggregation in response to drought has been found to amplify both WNV and St. Louis encephalitis virus in the Southeastern US [112]. Alternating periods of drought and drought-rebound have also been proposed as a mechanism driving WNV epidemics [113,114]. In the drought and drought-rebound scenarios, drought first produces elevated WNV prevalence during vector and host aggregation at limited water sources, and then drought-rebound facilitates virus geographic spread as vectors and hosts disperse to take advantage of newly available habitat created through augmented precipitation.

Network analysis of the WNV enzootic system indicated that WND risk was more closely associated with avian host phylogenetic community structure than climatic, land cover, or human demographic factors (Figure 1). Phylogenetic community structure is an indicator of disease pressure [115,116,117,118] and may have implications for WNV transmission. If avian species traits connected to virus transmission are evolutionary conserved, assemblages composed of species with high phylogenetic relatedness may provide greater opportunity for virus sharing, host switching, and spillover. Phylogenetic conservatism postulates that the degree of similarity among species traits, behaviors, and niches is correlated to the degree of relatedness between those species [119]. From this perspective, over-dispersion (lower than expected relatedness among co-occurring species) may be evidence of competitively structured communities whereas phylogenetic clustering (higher than expected relatedness among co-occurring species) may be indicative of environmental filtering [57]. That is, areas of over-dispersion identified by our analysis may signify the occurrence of avian assemblages with greater diversity in terms of both species richness and physiological traits than is expected based on a random draw from the species pool. By comparison, clustered communities might represent assemblages with greater relatedness and more comparable environmental tolerances, physiological characteristics, and life histories than random. Because birds are highly mobile, the general pattern shown by host eco-phylogenetic metrics in our study was one of relative phylogenetic over-dispersion in more southerly and coastal US locations during cooler months (November–March) with comparative clustering across the majority of mid-western and western states. This pattern changed during warmer months (April–October) to show less overall clustering and greater over-dispersion nationwide (Figure 4 and Figure A4, Figure A5, Figure A6, Figure A7, Figure A8, Figure A9, Figure A10, Figure A11, Figure A12, Figure A13, Figure A14, Figure A15). The spatiotemporal transitions observed between areas of over-dispersion and clustering tracked seasonal bird migration patterns in the US. During winter months, many passerine species relocate to either southern portions of the US or its thermally buffered coasts to avoid low-temperature extremes and ground-covering snowfall, whereas, non-migratory species in the clade typically possess metabolic traits that make them resistant to winter climate [120]. Thus, winter onset increases species richness at overwintering grounds in the south and along coasts (i.e., migratory bird influx) while concurrently filtering non-migratory species by cold-tolerance capacity (environmental filtering). The reverse process occurs as warmer spring and summer seasons approach, resulting in increased richness and trait diversity over the terrestrial US as a whole. In support of this interpretation, network analysis (Figure 1) and mapped community metrics (Figure 4 vs. Figure A4) show strong, positive correlation between species richness and phylogenetic distance.

When applied as WND predictive measures (Figure 5), species richness, phylogenetic distance, and mean nearest taxon were found to positively correlate to outbreak season timing (Dynamic versions exhibit positive polarity), but negatively associate with disease risk spatial distributions during the primary season (Static versions exhibit negative polarity). This signified that avian community composition temporally trended towards becoming less-related (more diverse, more phylogenetically distant) as WND risk increased moving into the outbreak season, but as diversity increased at a geographic location, concomitant disease risk proportionately decreased. In broader ecological terms, return migration from overwintering areas in the spring increased avian diversity across major portions of the US and migration timing largely coincided with the onset of the West Nile outbreak season; however, although the timing of these events was similar, locations with elevated diversity experienced lower WNV transmission rates than did less-diverse locations. Unlike phylogenetic relatedness measures, host (dynamic) molecular prevalence was negatively correlated to outbreak season timing, suggesting that average community virus competency decreased as host diversity and disease risk increased. Molecular prevalence estimated using a seasonal species pool (Static version) was not a statistically important predictor of WND risk, but consistent with other host metrics, exhibited an opposite polarity than that for the monthly varying species pool. The dynamic and static varieties of evolutionary distinctiveness and mean pairwise taxa were weak or insignificant disease risk indicators. However, it is worth noting that these measures showed contrasting polarity to species richness, phylogenetic distance, and Mean nearest taxon, which matches the expectation given that these measures may reflect host species competition [121]. In its totality, our analysis of host eco-phylogenetics suggested that avian species diversity is strongly associated with species migration, amplifies WNV prevalence in the time dimension, and effectively dilutes the virus in geographic space.

Our study faced two major data limitations. First, the ArboNET data that served as a basis for analysis were voluntarily provided to the CDC by counties. As voluntary data, case counts and virus detections were subject to county-level surveillance, collection, and reporting biases. Although our temporally and spatially-explicit model framework aided in accounting for many data biases, it was likely not perfect. Second, data reflecting equine WNV vaccination practices are not systematically collected in the US and were therefore not available for model inclusion. We suspect that vaccination practices may explain some of the WND variation revealed in our study, but controlling for this variation is problematic in the absence of detailed vaccination information. We did choose to assess the influence of household income as a potential proxy of vaccination, under the hypothesis that horses located in relatively high income areas might be more protected from WND due to owners being able to afford more consistent vaccination. Although we found that WND risk decreased in areas with increased income (Table 1), the linkage between household income and horse vaccination rates remains speculative in the absence of additional data.

## 5. Conclusions

Since initial invasion by WNV in 1999, approximately 30,000 horses in the US have been affected by neurologic disease and hundreds more are infected by the virus each year. Because of this, research elucidating the drivers of equine WND is greatly needed to better anticipate the spatial and temporal distribution of disease risk, improve disease surveillance, and avoid future economic impacts to the equine industry and private horse owners. To help meet this need, we applied a disease biogeography perspective and evaluated spatiotemporal relationships among four components of the WNV system: horse hosts, mosquito vectors, avian reservoirs, and climatic and demographic factors. Our findings pinpointed the timing and location of several high-risk WND areas throughout the US and can be used to prioritize virus surveillance and monitoring. Our analysis identified drought as a potential mechanism for virus amplification and demonstrated the importance of accounting for spatial non-stationarity when quantifying interaction between disease risk and meteorological influences such as temperature and precipitation. We also quantified avian host community dynamics across a massive geographic scale to show intra-annual variability in host phylogenetic structure and demonstrate host phylodiversity as a mechanism for virus amplification in time and virus dilution in space. Lastly, we encourage other researchers to expand on our methods for improved understanding of disease systems and to work towards fuller integration of spatiotemporal epidemiology, eco-phylogenetics, and distributional ecology.

## Figures and Tables

**Table 1:** Estimated coefficients for equine WND. Mean, standard deviation (SD) and 95% Credible Intervals. Coefficients are on the log scale with covariates judged to be significant based on credible intervals shown in bold text.

Covariate	Mean	SD	2.5 Q	97.5 Q
**WNV Surveillance**	0.14	0.03	0.09	0.20
Human Population Density	−0.03	0.03	−0.09	0.02
**Median Household Income**	−0.12	0.04	−0.19	−0.05
Population in Poverty (%)	−0.02	0.03	−0.05	0.01
Evergreen/Deciduous Needleleaf Trees	−0.09	0.05	−0.20	0.01
Evergreen Broadleaf Trees	−0.01	0.05	−0.10	0.09
Deciduous Broadleaf Trees	−0.16	0.10	−0.35	0.04
**Mixed/Other Trees**	−0.20	0.09	−0.38	−0.02
Shrubs	0.05	0.06	−0.07	0.16
Herbaceous Vegetation	0.02	0.08	−0.14	0.18
Cultivated and Managed Vegetation	0.02	0.10	−0.19	0.22
Regularly Flooded Vegetation	0.02	0.05	−0.08	0.11
**Urban/Built-Up**	−0.12	0.05	−0.21	−0.03
Snow/Ice	−0.02	0.02	−0.07	0.02
**Barren**	0.07	0.03	0.01	0.13
**Open Water**	−0.16	0.05	−0.25	−0.06
Elevation	−0.11	0.08	−0.26	0.05

## Data Availability

All disease occurrence and environmental data used in this study are freely available and can be accessed using the hyperlinks provided in Materials and Methods. Derived covariates for West Nile virus detection probability and avian host community phylogenetics are provided in the Appendix A.

## Abbreviations

The following abbreviations are used in this manuscript:

CDC	Centers for Disease Control and Prevention
RR	Relative Risk
SIR	Standardized Incidence Rate
SVC	Spatially varying coefficients
US	United States of America
WNV	West Nile virus
WND	West Nile disease
